# Does mass chloroquine treatment have any role in the elimination of *Plasmodium vivax* ?

**DOI:** 10.1186/s12936-025-05399-2

**Published:** 2025-05-26

**Authors:** Nicholas J. White, Somya Mehra, James A. Watson

**Affiliations:** 1 Mahidol Oxford Tropical Medicine Research Unit, Mahidol University, Bangkok, 10400 Thailand; 2 Centre for Tropical Medicine and Global Health, University of Oxford, Oxford, OX3 7LG UK; 3 Infectious Diseases Data Observatory, Oxford, UK

**Keywords:** *Plasmodium vivax*, Chloroquine, Mass drug administration, Elimination

## Abstract

Countries in the Greater Mekong sub-region (GMS) have been encouraged to deploy mass chloroquine treatments given monthly for four months to reduce the burden of vivax malaria. This paper summarizes briefly current knowledge on *Plasmodium vivax* epidemiology, the biology of vivax relapse and previous experience using dihydroartemisinin–piperaquine mass treatments in the GMS to show why this approach would be extremely cost-ineffective. Around 800 full treatment courses in 200 people would be needed to prevent one symptomatic case. Mass chloroquine treatment will contribute little or nothing to the elimination of vivax malaria in this area.

**Supplementary Information:**

The online version contains supplementary material available at 10.1186/s12936-025-05399-2.

## Introduction

*Plasmodium vivax* is now the main cause of malaria outside Africa. *P. vivax* is more difficult to eliminate than *Plasmodium falciparum* because of relapses which arise from the activation of dormant liver stage parasites (“hypnozoites”). In areas with low malaria transmission and efficient and effective case management, a combination of vector control and efficacious blood stage drug treatments can drive *P. vivax* slowly to elimination. This happened in Europe, the former Soviet republics, China, North Africa, the Middle East and North America. These elimination initiatives in the past 70 years were variably augmented by radical cure of acute malaria with an 8-aminoquinoline antimalarial (usually primaquine), and in some areas by mass primaquine treatments [1]. The 8-aminoquinoline treatment is needed to eliminate the persistent liver stages (hypnozoites) which cause relapses. This is called “radical cure”. In vivax malaria treatment chloroquine or an artemisinin-based combination treatment is used to treat the acute malaria episode (active against blood stage parasites only), and the 8-aminoquinoline is added to provide the radical cure. Unfortunately radical cure cannot be given to everyone who needs it. All the 8-aminoquinolines carry the risk of causing oxidant haemolysis in patients who are glucose-6-phosphate dehydrogenase (G6PD) deficient, and these drugs cannot be taken in pregnancy.

The predominant form of *P. vivax* prevalent today is the short incubation period, frequent relapse, tropical phenotype typified by the Chesson strain [2]. The long incubation or long latency phenotypes (typified by the Madagascar or St Elizabeth strains) which predominated in North America, Europe and Northern Asia, are now a relative minority [2, 3]. In the Greater Mekong subregion (GMS) of Southeast Asia *P. falciparum* and *P. vivax* have historically each comprised about half the symptomatic malaria infections. Recent malaria elimination initiatives based mainly on provision of rapid diagnostic tests and effective antimalarial drugs to village health workers together with strengthening of other control activities, and in some areas mass treatments to eliminate *P. falciparum*, have led to a marked decline in malaria incidence—particularly in the Eastern GMS, where human malaria is now very close to being eliminated [4–7]. In contrast, in the western GMS, these substantial gains are reversing consequent upon disruption caused by the civil war in Myanmar. Throughout the region *P. falciparum* has declined faster than *P. vivax* (which has therefore become the predominant malaria).

To reduce the burden of *P. vivax* in the GMS and progress towards the elimination of all human malaria in the region the World Health Organization (WHO) in this region has recently been encouraging countries to conduct serial mass treatments over four months with chloroquine (see recommendation 6 in Sect. 3.2.1 in [7]). There is no evidence to support this recommendation. This paper reviews the current malaria epidemiology in the GMS region and the relevant biology of vivax malaria to estimate the number of mass chloroquine treatments and the number of people who would need to receive these treatments (i.e., number needed to treat: NNT) in order to prevent one symptomatic case. The WHO’s estimates, recent epidemiological data from studies of mass dihydroartemisinin-piperaquine treatments, and mathematical modelling of detailed malariometric data from the western border of Thailand were used to evaluate the conditions under which mass chloroquine treatments could eliminate *P. vivax* malaria in the GMS region.

### Malaria epidemiology in the GMS

Malaria in the GMS is largely a disease of remote hill forested areas. The main anopheline vectors are *Anopheles dirus* complex, *Anopheles minimus* and *Anopheles maculatus*. Malaria is predominantly a rainy season disease. There are commonly two incidence peaks—one at the beginning and one just after the rains. Malaria occurs at all ages as transmission intensities overall are typically low throughout the region (*P. vivax* entomological inoculation rate (EIR) is usually < 1/year). At a greater granularity, transmission intensities are distributed irregularly with substantial variation over short distances and small hot-spots of higher transmission. *Plasmodium vivax* prevalence varies correspondingly with the majority of carriage being asymptomatic and below the level of microscopy detection (the geometric mean blood density is approximately 5000 parasites/mL). Village populations typically comprise 500–1300 people. There is often substantial people movement. Younger males comprise a high proportion of reported clinical cases although, within a transmission focus among the resident population, vivax malaria is predominantly a disease of children. *Plasmodium vivax* in the GMS is still relatively chloroquine sensitive although there is some evidence for reduced susceptibility [8]. *Plasmodium vivax* relapses with short intervals between episodes—typically three weeks after treatment with a rapidly eliminated drug such as artesunate or quinine and 6–7 weeks after chloroquine or piperaquine [2, 9]. The longer interval to relapse following chloroquine results from suppression for several weeks of the blood stage infection which, following hypnozoite activation, emerges from the liver about two weeks after the acute malaria episode. Radical cure with primaquine is increasingly used for radical cure in the GMS, although G6PD testing is still not available widely.

### Mass chloroquine treatment to reduce the burden of vivax malaria in the GMS

A recent WHO convened workshop on malaria surveillance and malaria elimination in the Greater Mekong Subregion (November 2023) recommended deploying four monthly rounds of chloroquine mass treatments in Thailand, Vietnam, Cambodia, and Lao PDR to reduce the burden of vivax malaria [7]. *Plasmodium vivax* asymptomatic carriage rates typically average around 7% in villages with > 5 cases/500 population per year, but can be as high as 20% in malaria hot-spots. Deploying four rounds of chloroquine MDA and assuming this will prevent half the incident cases (i.e. 100% adherence and assuming that half the annual incident cases occur during the 4 month MDA period) for an average village population of 500 persons and 5 incident cases/year results in approximately 800 administered treatments per prevented case (i.e., 4 × 500 treatments to prevent 0*.*5 × 5 cases). Thus, assuming 100% adherence, the number of people who need to be treated with 4 monthly rounds of chloroquine treatment to prevent one case of symptomatic vivax malaria is 200. This number rises as the prevalence falls. Chloroquine is inexpensive, but organizing repeated malaria MDA in remote areas is not.

### Mass treatment experience with dihydroartemisinin–piperaquine

**Fig. 1:**
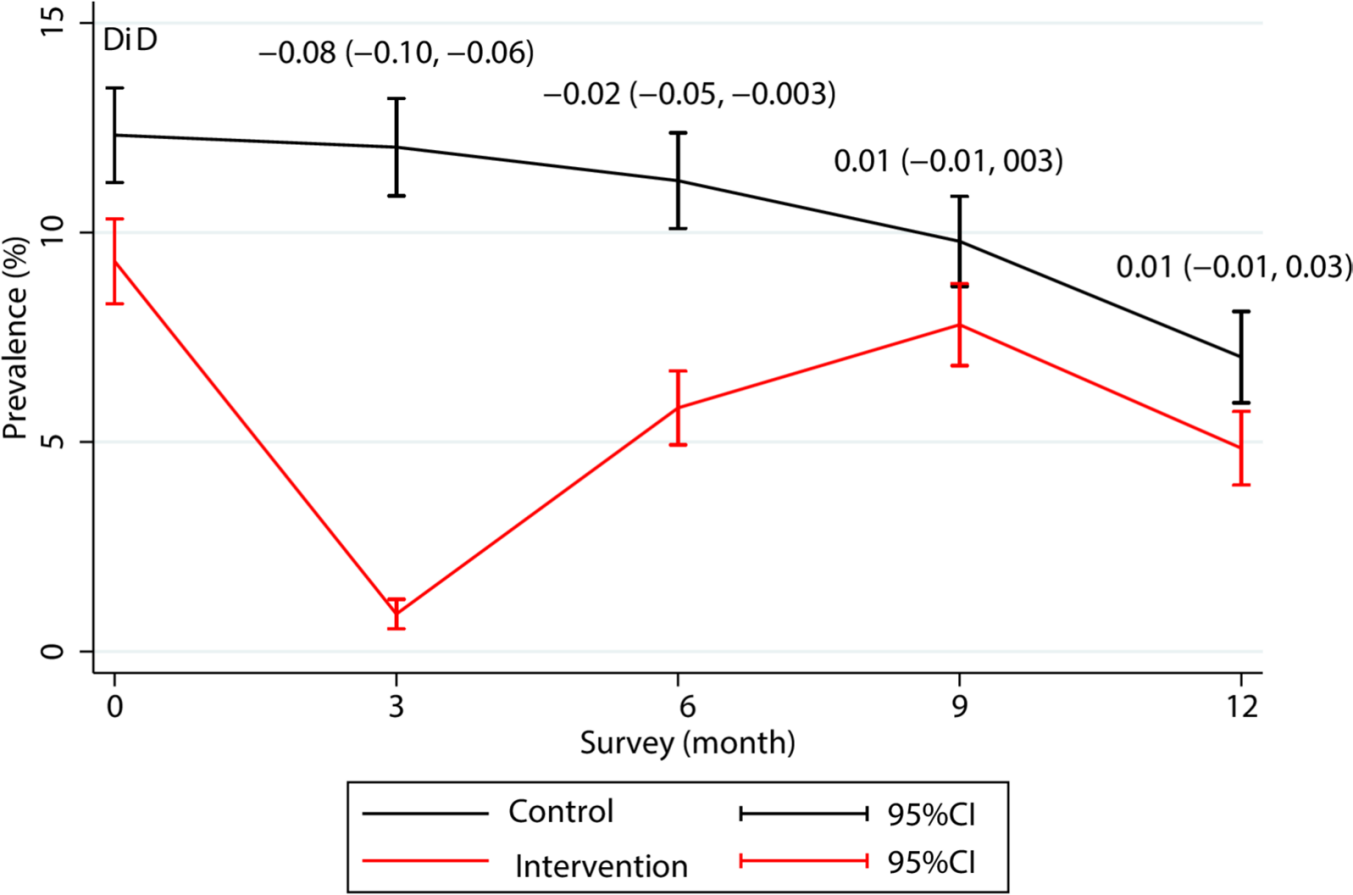
Changes in the prevalence of *P. vivax* infection during 12-month follow-up in control and dihydroartemisinin–piperaquine MDA intervention villages in a study conducted in Eastern Myanmar (reproduced from [11]). The prevalence of vivax malaria in one village with a low initial prevalence remained lower after MDA intervention when compared to baseline values (2% vs 14%). This suggested that under some epidemiological conditions mass blood stage treatment could have a more lasting effect, but concluding causality is not possible

To accelerate the elimination of falciparum malaria and thereby counter the threat of artemisinin resistance in the GMS, mass treatments (MDAs) with dihydroartemisinin–piperaquine (DP) and single low dose primaquine were evaluated in prospective studies. These studies began in Eastern Myanmar in 2013. Piperaquine, a bisquinoline compound, results in slightly longer post-treatment prophylaxis (6–7 weeks) compared to chloroquine but otherwise has very similar pharmacokinetic and pharmacodynamic properties. The prospective MDA studies in the GMS region were preceded by epidemiological studies to characterize the prevalence of sub-microscopic malaria infections and to identify “hot-spots” of much higher transmission. Together with strengthened support for village malaria workers, these DP MDAs given in three rounds at one month intervals, proved well tolerated and effective in contributing to the elimination of *P. falciparum*, but in some areas *P. vivax* very soon returned [10] (Fig. 1). For example, in a detailed epidemiological and entomological evaluation of dihydroartemisinin–piperaquine MDA conducted in Eastern Myanmar, the prevalence of asymptomatic [11] vivax malaria ranged from 12 to 31% before MDA and fell to < 1*.*6% in all villages during MDA []. This suggested that the MDA was associated with a 12.5-fold decrease (95% CI 1.6–100) in the *P. vivax* EIR. However, in that area the reservoir of asymptomatic vivax infections reconstituted in the three months following MDA (attributed to relapse from hypnozoites as no radical cure regimens were used). This was coincident with a 5.5-fold increase (95% CI, 4.0–6.3) in the prevalence of *P. vivax* –infected vectors.

### Modelling the hypnozoite burden following chloroquine MDA

**Fig. 2:**
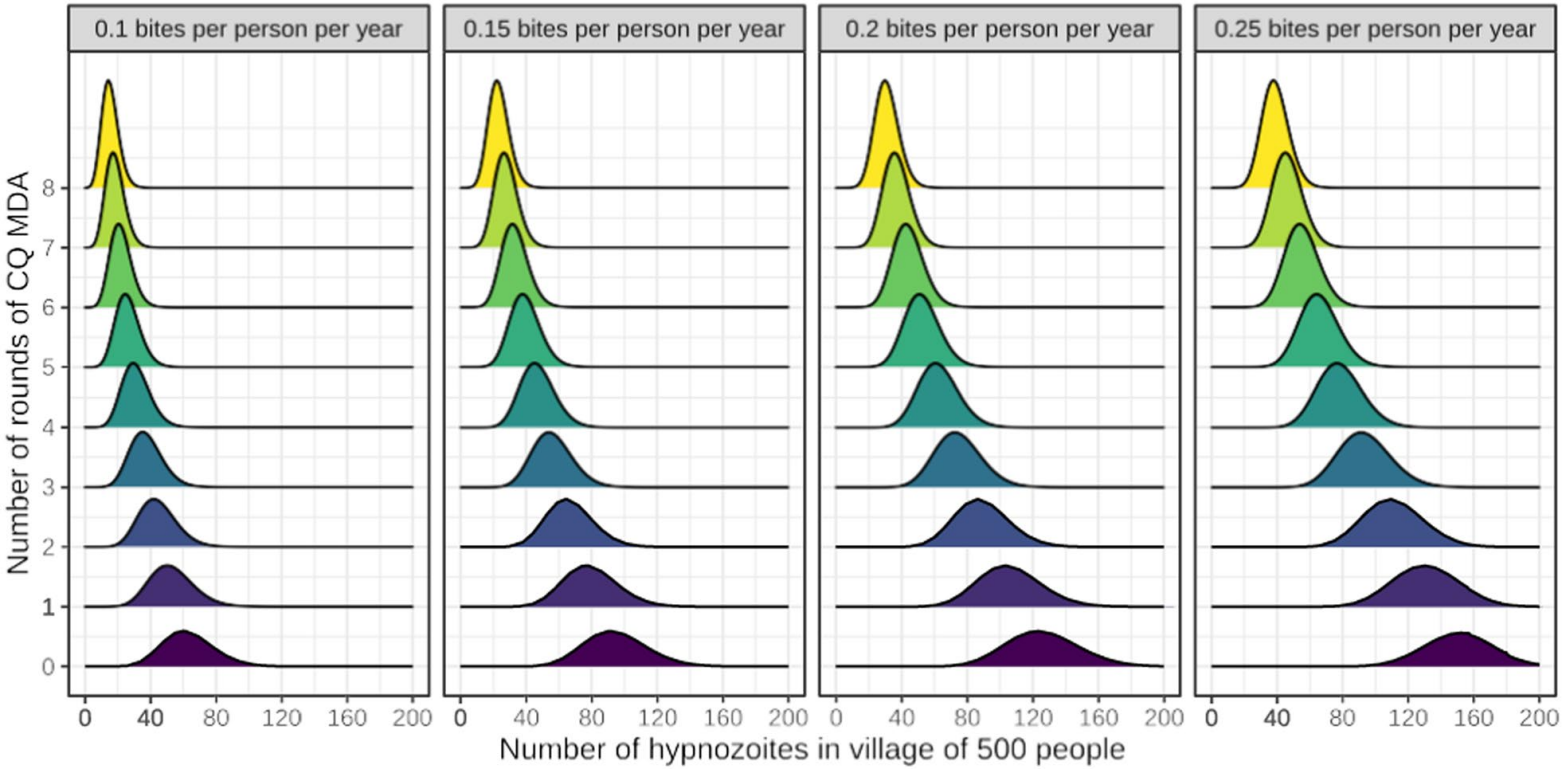
Hypnozoite burdens, summed over a village of 500 people, before and after successive rounds of chloroquine MDA based on [15]. Panels correspond to the population-average EIR, whereby 50% of bites are concentrated in 20% of individuals. Model assumptions and derivations are detailed in the Supplementary material

Detailed parasitological data from malaria therapy, volunteer studies and more recently from field epidemiology studies provides information on the temporal patterns of *P. vivax* hypnozoite activation. These observations and deductions allow prediction of the impact of malaria control interventions under different conditions (transmission intensity, seasonality, efficacy, adherence etc.). A model of hypnozoite latency periods based on independent random activation has previously been developed [12, 13]. Calibration of the model to a highly detailed prospective cohort study of *P. vivax* infections (conducted on the Thailand-Myanmar border over 30 years ago) [14], suggests an average *P. vivax* hypnozoite elimination half-life of four months, with an average of 3 hypnozoites established per *P. vivax* infected anopheline bite [15]. This model is used to characterise the steady state hypnozoite burden as a function of the EIR (see Supplementary material). For illustration, Fig. 2 shows the steady state hypnozoite burden summed over a village of 500 people and adjusted for population heterogeneity such that the 50% of infective bites are concentrated in 20% of human hosts.

Chloroquine is efficacious against the blood stages of *Plasmodium vivax* but it does not affect either the hypnozoites or pre-erythrocytic development within hepatocytes. If the average hypnozoite elimination half-life is four months, as our recent epidemiological analyses suggest [15], then a four month period of mass chloroquine treatment (25 mg base/kg over 3 days at monthly intervals) on average will leave behind approximately half the hypnozoites that were present before the MDAs. By the same reasoning eight rounds of MDA would leave behind approximately a quarter of the pre-MDA hypnozoites (Fig. 2).

The incremental advantage in terms of accelerated *P. vivax* elimination from giving mass chloroquine is relatively small. For example, assuming 100% adherence, if four rounds of chloroquine MDA were administered in a village of 500 people in which each person receives a *P. vivax* infected anopheline mosquito bite once every 10 years on average, this would leave behind approximately 30 hypnozoites. After the MDAs, these hypnozoites would likely activate to reinstate onward transmission.

Where the force of infection (the number of malaria infected anopheline mosquito bites received per person per year) is very low, malaria may die out naturally as a result of stochastic processes. The thresholds for elimination will depend on the distribution of infections within people. These distributions tend to be highly overdispersed with a minority of the population harbouring the majority of infections. Elimination of vivax malaria relies upon there being no reintroduction from adjacent areas of more intense transmission. It also depends on the proportion and duration of asymptomatic blood stage infections derived from emerging hypnozoites. Taken together, this evidence suggests that the only conditions under which chloroquine mass treatments given over four months can help to eliminate vivax malaria are in the situations where transmission is so low that spontaneous elimination could occur anyway. Where there is a higher force of infection, mass chloroquine treatments given for four months cannot eliminate vivax malaria.

## Discussion

Using mass chloroquine treatment to reduce the *P. vivax* burden in low transmission settings is extremely inefficient. In the current scenario up to 800 treatment courses given to 200 people are needed to prevent a single symptomatic case of vivax malaria. This substantial effort would be justified only if there is a reasonable prospect of eliminating *P. vivax*. Relapse is the main obstacle to *P. vivax* elimination. Clinical, epidemiological and parasitological evidence suggest that *P. vivax* hypnozoites, even in the tropical frequent relapse strains, are relatively long lived. Fitting a mathematical model to prospectively gathered serial data obtained on the Thailand-Myanmar border suggests an approximate hypnozoite half-life of four months [15]. As chloroquine does not kill hypnozoites or prevent pre-erythrocytic development, administering chloroquine, or any other suppressive prophylaxis, for four months will leave behind about half the pre-MDA “steady state” hypnozoite burden. Hypnozoites which activate more than two weeks after the final chloroquine dose will generate asexual stage parasites which can survive the declining residual drug concentrations and reach patent transmissible parasite densities [16]. Thus small numbers of hypnozoites in the overall population will still survive the four months chemoprevention and may prevent elimination of *P. vivax*. Vivax malaria will return rapidly, just as it did after the dihydroartemisinin–piperaquine MDAs used to accelerate *P. falciparum* elimination [11]. Four month chloroquine MDAs therefore will not eliminate vixax malaria under most transmission conditions.

If the mass treatments with chloroquine are continued for even longer periods (effectively providing continuous chemoprophylaxis) the numbers of residual hypnozoites will decline as transmission is interrupted and hypnozoites spontaneously activate. Eventually, provided adherence is good, in theory vivax malaria can be eliminated. The idea that continuous suppressive prophylaxis could eliminate malaria was the rationale behind the ill-fated Pinotti method of adding chloroquine (and pyrimethamine) to table salt. The Pinotti method did not work, and is credited with selecting for chloroquine resistance in *P. falciparum* [17]. The chance of eliminating all *P. vivax* infections therefore depends on the pre-existing hypnozoite burdens, their distribution within the human population, the duration and efficacy of the suppressive prophylaxis, the village population sizes, population movement (re-seeding from neighboring areas), and population adherence to the repeated mass treatments. The logistic requirements of deploying mass drug administration effectively in remote areas are substantial. In practice, the expensive and demanding intervention of providing chloroquine MDA for four months will not eliminate vivax malaria in the areas where it is currently being advocated.

Malaria has been reduced substantially in the Eastern Greater Mekong subregion. In January 2024 Cambodia reported 22 cases in the entire country and Lao PDR reported 39 [18]. If these figures are correct then transmission is already so low in some areas that malaria may eliminate spontaneously. There is no justification for a costly mass treatment campaign to provide a marginal acceleration. In the few higher transmission foci the residual hypnozoite reservoir will prevent elimination by mass chloroquine treatment.

The recent experience of evaluating dihydroartemisinin–piperaquine for mass treatment as a *P. falciparum* elimination accelerator provides a clear comparative case study. These three month mass treatments were effective in accelerating *P. falciparum* elimination but *P. vivax* returned rapidly, measured both in the anopheline vectors and in the resident human population [11]. This was attributed to residual hypnozoite activation. Mass treatment with chloroquine or any other similar drug (e.g., piperaquine) will only eliminate *P. vivax* if the existing hypnozoite burden is very low and the mass treatments are continued for many months. This is highly unlikely to be cost effective as large numbers of people without asymptomatic *P. vixax* carriage and with no hypnozoites will receive repeated chloroquine treatment doses. Indeed, aside from the difficulties in ensuring high levels of adherence, it is debatable whether there are any circumstances at all in which mass chloroquine is a cost-effective vivax malaria elimination measure.

## Supplementary Information

Supplementary Material 1.

## Data Availability

No datasets were generated or analysed during the current study.
