# Supplementary material for: Does mass chloroquine treatment have any role in the elimination of Plasmodium vivax ?
Source: Malar J. 2025 May 26;24:166. doi: 10.1186/s12936-025-05399-2 (PMC12105338; doi:10.1186/s12936-025-05399-2)
Supplement: Supplementary file 1 — Supplementary Material 1. [file 12936_2025_5399_MOESM1_ESM.pdf]

Supplementary information. To model the hypnozoite burden, we draw on the within-host framework constructed in [15]. We adopt the simplest exponential clock model, whereby each hypnozoite activates independently at constant rate η [13, 15]. Each infective bite is modelled to inoculate a geometrically-distributed sporozoite batch of mean size ν. A sporozoite either undergoes immediate development with probability $(1 - p_{\text{rel}})$ to give rise to primary infection or it forms a hypnozoite that is destined to activate at a later time (we have re-parametrised this model to ignore the unobservable contribution of hypnozoite death). To capture population heterogeneity, we allow the force of inoculation in the population to follow a Gamma-distribution, with shape parameter κ and mean $\bar{\lambda}$ [19].

Under a constant force of infection or infective bite rate λ, the hypnozoite burden at stationarity follows a negative binomial distribution with mean $\lambda\nu p_{\text{rel}}/\eta$ and shape parameter λ/η [12]. Each round of chloroquine MDA is assumed to confer T days of chemoprotection. If an individual receives m rounds of chloroquine, this amounts to a consecutive duration mT of complete prophylactic antimalarial protection. During this period, each hypnozoite may activate spontaneously with probability $(1 - e^{-\lambda mT})$, but the hypnozoite progeny will be unable to establish a blood-stage infection. Assuming that additional hypnozoites cannot be acquired during this period of chemoprotection (i.e. that transmission of *P. vivax* has also been interrupted completely), we can apply the law of total expectation to show that the size of the hypnozoite reservoir $H_m(\lambda)$ following m bouts of treatment is negative binomial with mean $\lambda\mu e^{-\eta mT}/\eta$ and shape parameter λ/η.

By applying the law of total expectation, we can show that the within-host hypnozoite burden H_m after m bouts of treatment, accommodating heterogeneity, has probability generating function (PGF)

$$\mathbb{E}\big[z^{H_m}\big] = \int_0^\infty \underbrace{\left(1 + \nu p_{\text{rel}} e^{-\eta mT}(1-z)\right)^{-\frac{u}{\eta}}}_{\substack{\text{PGF at stationarity} \\ \text{under constant FOI } u}} \cdot \underbrace{\frac{1}{\Gamma(\kappa)}\left(\frac{\kappa}{\bar{\lambda}}\right)^{\kappa} u^{\kappa-1} e^{-\frac{\kappa u}{\bar{\lambda}}}}_{\substack{\text{probability density} \\ \text{(Gamma distribution) for FOI } u}} \, du$$

$$= \left(1 + \frac{\bar{\lambda}}{\eta\kappa} \log\left(1 + \nu p_{\text{rel}} e^{-\eta mT}(1-z)\right)\right)^{-\kappa}.$$

Now, we consider a closed population of N individuals with 100% adherence to an MDA regimen spanning m bouts of treatment. Assuming that the hypnozoite burden of each individual can be modelled independently, the population-wise hypnozoite burden $H_m(N)$ after MDA has PGF

$$\mathbb{E}\big[z^{H_m(N)}\big] = \mathbb{E}\big[z^{H_m}\big]^N = \left(1 + \frac{\bar{\lambda}}{\eta\kappa} \log\left(1 + \nu p_{\text{rel}} e^{-\eta mT}(1-z)\right)\right)^{-\kappa N}. \tag{1}$$

We can recover the probability mass function (PMF) of $H_m(N)$ from the PGF (1) by computing

$$P\big(H_m(N) = n\big) = \frac{1}{n!}\frac{d^n}{dz^n}\mathbb{E}\big[z^{H_m(N)}\big]\bigg|_{z=0}.$$

This can be performed directly using the function `Series` in Mathematica. To obtain an analytic expression, we write the PGF (1) in the form

$$\mathbb{E}\big[z^{H_m(N)}\big] = f(g(z))$$

where

$$\begin{aligned} f(z) &= x^{-\kappa N} \\ g(z) &= 1 + \frac{\bar{\lambda}}{\eta\kappa}\log\big(1 + \nu p_{\text{rel}} e^{-\eta m T}(1-z)\big). \end{aligned}$$

Using Faa di Bruno's formula as in [12], it then follows that

$$\begin{aligned} P\big(H_m(N) = n\big) &= \frac{1}{n!}\sum_{\ell=0}^{n} f^{(\ell)}(g(0)) \cdot B_{n,\ell}\big(g^{(1)}(0), \dots, g^{(n-\ell+1)}(0)\big) \\ &= \sum_{\ell=0}^{n}(-1)^{\ell}\frac{\ell!}{n!}\binom{\kappa N + \ell - 1}{\ell}\left[1 + \frac{\bar{\lambda}}{\eta\kappa}\log(1 + \nu p_{\text{rel}} e^{-\eta m T})\right]^{-(\kappa N + \ell)} \\ &\qquad B_{n,\ell}\left(-\frac{\bar{\lambda}}{\eta\kappa}\frac{\nu p_{\text{rel}} e^{-\eta m T}}{\nu p_{\text{rel}} e^{-\eta m T} + 1}, \dots - \frac{\bar{\lambda}}{\eta\kappa}(n-\ell)!\left(\frac{\nu p_{\text{rel}} e^{-\eta m T}}{\nu p_{\text{rel}} e^{-\eta m T} + 1}\right)^{n-\ell+1}\right) \end{aligned}$$

where $B_{n,k}$ denotes the partial Bell polynomial. Using standard identities, we obtain

$$\begin{aligned} P\big(H_m(N) = n\big) = \sum_{\ell=0}^{n}\Bigg\{ &\frac{\ell!}{n!}\begin{bmatrix} n \\ \ell \end{bmatrix}\binom{\kappa N + \ell - 1}{\ell}\left(\frac{\bar{\lambda}}{\eta\kappa}\right)^{\ell}\left(\frac{\nu p_{\text{rel}} e^{-\eta m T}}{\nu p_{\text{rel}} e^{-\eta m T} + 1}\right)^{n} \\ &\left[1 + \frac{\bar{\lambda}}{\eta\kappa}\log(1 + \nu p_{\text{rel}} e^{-\eta m T})\right]^{-(\kappa N + \ell)}\Bigg\} \end{aligned}$$

where $\begin{bmatrix} n \\ \ell \end{bmatrix}$ denotes an unsigned Stirling number of the first kind.

We parametrise this model based on the posterior median estimates $\eta = 1/171$ day^{-1} and $\nu p_{\text{rel}} = 2.7$ from [15], with $\kappa = 1.15$ such that the 20% of individuals subject to the highest transmission intensity are collectively expected to experience 50% of infective bites. We assume each round of CQ MDA yields $T = 30$ days of chemoprotection.
